# Emotional Intelligence and Attention-deficit/hyperactivity disorder (ADHD)

**DOI:** 10.1192/j.eurpsy.2023.1087

**Published:** 2023-07-19

**Authors:** I. Ben Turkia, T. Brahim, L. Sahli

**Affiliations:** child and adolescent psychiatry department, Rouvray Hospital center, Sotteville-les-Rouen, France

## Abstract

**Introduction:**

Attention-deficit/hyperactivity disorder (ADHD) is the most common neurodevelopmental disorder in children and adolescents. It is characterized by age-inappropriate inattention/impulsiveness and/or hyperactivity symptoms.

However, emotional symptoms are frequent in patients with ADHD and may, in some cases, be responsible for a major part of the negative impact on functioning and outcome.

It is now well established that a large number of children with ADHD and without any comorbid disorder exhibit symptoms of emotional lability.

Recently , given the importance of the impact of emotional symtoms , several authors have argued that emotional intelligence affects health and is essential for success in academics as well as life in general and it is defined as the ability to perceive, appraise, and express emotions; the ability to access and/or generate feelings when they facilitate thought; and the ability to regulate emotions to promote emotional and intellectual growth.

**Objectives:**

•Our research aimed to evaluate and compare the emotional and social functioning of two groups of children, with and without ADHD , aged 6 to 19 .

**Methods:**

•One hundred twenty child (N=60 ADHD, N = 60 Control cases) were assessed with the **BarOn Emotional Quotient Inventory: Youth Version (BarOn EQ-i:YV™),** providing an estimate of their underlying emotional and social intelligence.

•The BarOn EQ-i:YV is specifically designed to assess the coping skills, adaptability, and well-being of children and teenagers.

•Children with ADHD and control cases were compared with each other.

**Results:**

•The results showed that the Emotional Quotient (EQ) was significantly lower in the group of children with ADHD **(p=0.01)**.

•Also, our results showed that there are statistically significant differences in **intrapersonal skills** (p˂0.0010) **Adaptability Scale**(p=0.005) ; **General Mood Scale (**p=0.004) and **positive impression** (p = 0.001) of emotional intelligence between children with ADHD and control cases. Thus, the first group got lower scores than the second one in all aspects.

**Conclusions:**

•ADHD is a disorder that affects the life quality of the person who suffers from it in the personal and social areas. Therefore, the emotional intelligence study in individuals with this diagnosis is important.

•And considering the fact that abilities associated with emotional intelligence can be learned and improved, emotional intelligence can be thought as a target for therapy by individualized education for patients with ADHD who have inadequate abilities compared to the healthy population .

**Disclosure of Interest:**

None Declared

